# Dragonflies community assembly in artificial habitats: Glimpses from field and manipulative experiments

**DOI:** 10.1371/journal.pone.0214127

**Published:** 2019-06-21

**Authors:** Francesco Cerini, Marco A. Bologna, Leonardo Vignoli

**Affiliations:** Dipartimento di Scienze, Università Roma Tre, Viale G. Marconi, Roma, Italy; University of Sydney, AUSTRALIA

## Abstract

Several factors act on community structure, so determining species composition and abundance patterns. Core processes operating at local scales, such as species-environment matching and species interactions, shape observed assemblages. Artificial habitats (simplified structure) are useful systems for assessing the main factors affecting community composition and disentangling their assembly rules. Drinking troughs (brickwork tanks for free-ranging cattle watering) are widespread in Italy and represent a suitable aquatic habitat for colonization by various aquatic organisms. Dragonflies larvae are usually found in drinking troughs and often exhibit strong species interactions and striking community assembly patterns. Our primary aim was to search for Odonata communities exhibiting non-random co-occurrence/segregation patterns in drinking troughs. We performed null-model analyses by measuring a co-occurrence index (C-score) on larval Odonata assemblages (13 species from 28 distinct troughs). Overall, we found a non-random structure for the studied dragonfly assemblages, which, given their fast generation time, must have been generated by short-term ecological processes (i.e. interspecific interactions). We thus analyzed potential competition/predation among and within ecological guilds. From the field data, we speculated that interactions within the sprawlers’ guild is likely among the main drivers structuring the studied assemblages, especially the effect of intraguild predation between *C*. *erythraea* and *Sympetrum* spp larval stages. We then experimentally tested these interactions in laboratory and demonstrated that intraguild predation among larvae at different development stages may result in an effective exclusion/negative impact on density pattern, representing one of the processes to take into consideration when studying dragonfly assemblages.

## Introduction

The species found when surveying assemblages are the result of past and present ecological processes that shape the survival of organisms [1]. Several factors act on community structure at different levels, determining species composition and abundance patterns. Such patterns were interpreted by Diamond [2] as the results of what he called “assembly rules”. Since then, several ecologists have been searching for generalizable rules able to explain plant and animal community patterns, with the aim (once the rule has been stated) of predicting community change. We can simply define assembly rules as explicitly stated constraints on community structure that limit which species can belong to locally coexisting subsets of the defined regional species pool. The assemblages that conform to the rules have a greater likelihood of existence, while those that deviate more widely from the assembly rules may exist for shorter time periods but will probably be replaced by assemblages more closely conforming to the constraints over time [1].

Both regional and local factors are responsible for the observed species composition. At the regional scale, the distinct species pool and the degree of reciprocal isolation among satellite habitats from source environments affect species composition within local sites. These factors place an upper boundary on species richness by determining which and how many species arrive at sites suitable for local establishment [3]. However, there are also core processes acting at the local scale (i.e. community level), such as the species’ abiotic requirements and the species’ interactions (competition, predation, etc.). To identify and study such processes in nature is a hard task and often requires long term and expensive work.

Artificial habitats, due to their simplified structure compared to natural habitats, provide a useful model for assessing the main factors affecting community composition and disentangling their assembly rules. Indeed, the environmental variables of any artificial habitat are low in number (compared to natural conditions) and are often more stable (anthropic control), making it easier to study complex ecological processes such as species interactions. In Italy, troughs used for free-ranging cattle drinking represent a widespread and very simplified artificial aquatic habitat. Despite drinking troughs being characterized by a very short age as aquatic environment (i.e. at maximum 70 years), they represent a suitable habitat for colonization by a variety of invertebrates and vertebrates (i.e. mollusks, annelids, crustaceans, insects, amphibians) [4].

Odonata are usually found in drinking troughs in Italy and represent an interesting model for studying such patterns of assemblage structure due to their dispersal capabilities, to their complex life cycle (aquatic at the larval stage and terrestrial when adult), and, finally, to their well-studied behaviour together with their intra and inter-specific interactions [5]. Indeed, Odonata exhibits a good species diversity, with asynchronous development of some species and the seasonal variation in life history patterns. These traits can create a complex situation where interspecific and intraspecific interactions likely play a role in assemblage patterning [6]. The importance of such interactions in dragonflies is well known, especially at the larval stage, when mutual predation (often size-dependent) can deeply shape the structure of communities [7]. The very simplified habitat characterizing the drinking troughs (e.g. limited volume, simple vegetation structure and diversity) and the absence of predator (fish) should facilitate population growth to high density, at which the occurrence of interspecific negative interactions (competition/predation) are more likely to occur. The physical and ecological features of a site are known to affect which species arrive and stay [8]. However, drinking troughs’ physical parameters present low variability within the same region because these artificial habitats are built with a similar shape and size. Moreover, since inter-specific interactions in dragonflies are intense in fish-free habitats [9], the absence of fish in drinking troughs represent another suitable condition for revealing such processes.

Therefore, we focus on interspecific interactions that could arise from, and be exacerbated by, heterochronic sequence of drinking trough species colonization that may influence assemblage patterning [10] with early colonists affecting the establishment of later species by outcompeting them [11] and/or by preying on them. To distinguish a pattern of species segregation from a change in species composition driven by a gradient in habitat quality or by limited dispersal, the studied habitats should be identical in the replicate patches and there should be no limits to dispersal [12,13]. Again, the high physical similarity of drinking troughs and their dense distribution in free-ranging cattle farms (easily reachable by dragonflies for their high dispersal capability) limit potential biases related to gradient and dispersal issues. In short, the effects of ecological assembly rules of the Odonata community should be amplified and easily observable in artificial habitats such as drinking troughs.

To uncover non-random patterns of species co-occurrence/segregation, we compared the observed assemblage in drinking troughs to proper null models [1]. Artificial simplified habitats provide a very effective system for studying animal assemblage patterning because most shortcomings that affect such pattern-uncovering in natural systems here are marginal (i.e. data quality and resolution, and community definition). Different species belong to a same community if they interact is some ways on a shared site [14]. This means that the status of sympatry or syntopy is not sufficient to assign two species to the same community [15]. Among artificial habitats, drinking troughs, with their physical boundaries enclosing the whole community, and with their ease of sampling by standard methods enabling collection of all the species belonging to the community, provide a very useful scale-effective system for uncovering assemblage structure in animal communities. Moreover, in traditional rural environments, drinking troughs are usually widely spaced out on the landscape, and have been in place for a time long enough to enable the establishment of communities, thus representing a self-set replicated experimental system.

With this study we used both field and laboratory approaches to answer general and specific questions on Odonata community assembly patterns. Indeed, we integrated classic co-occurrence analysis with an experimental hypothesis-testing approach to address specific issues rising from simple inferences on aggregated or segregated community patterns (checkerboard distributions) [16]. In detail, we want to answer the following key questions: 1) Do Odonata communities exhibit non-random co-occurrence/segregation patterns in artificial habitats? 2) Are these patterns generated by habitat constraints, interspecific interactions, or stochastic processes? 3) Are there possible generalizable processes leading to assembly rules for predicting how Odonata communities would evolve in simplified habitats like those considered in this study?

## Materials and methods

### Study area

Our study area is located within the Special Protected Area (SPA) “Monti della Tolfa”, Central Italy (Lazio) and stretches for about 77000 hectares. It is bounded by the Tyrrhenian Sea coast on west, by the Sabatini Mountains on east, and by the Cimini Mountains on north. Tolfa and Allumiere are the principal towns. The main landscape is dominated by pastures devoted at sustaining free-range cattle. The total number of drinking troughs spread all over the area is nearly 200, reflecting scarcity of natural water bodies. No specific permissions were required for sampling in this area since it is not defined as Regional or National Reserve or Park. Our study did not involve any endangered or protected species.

### Sampling methods

**Fig 1:**
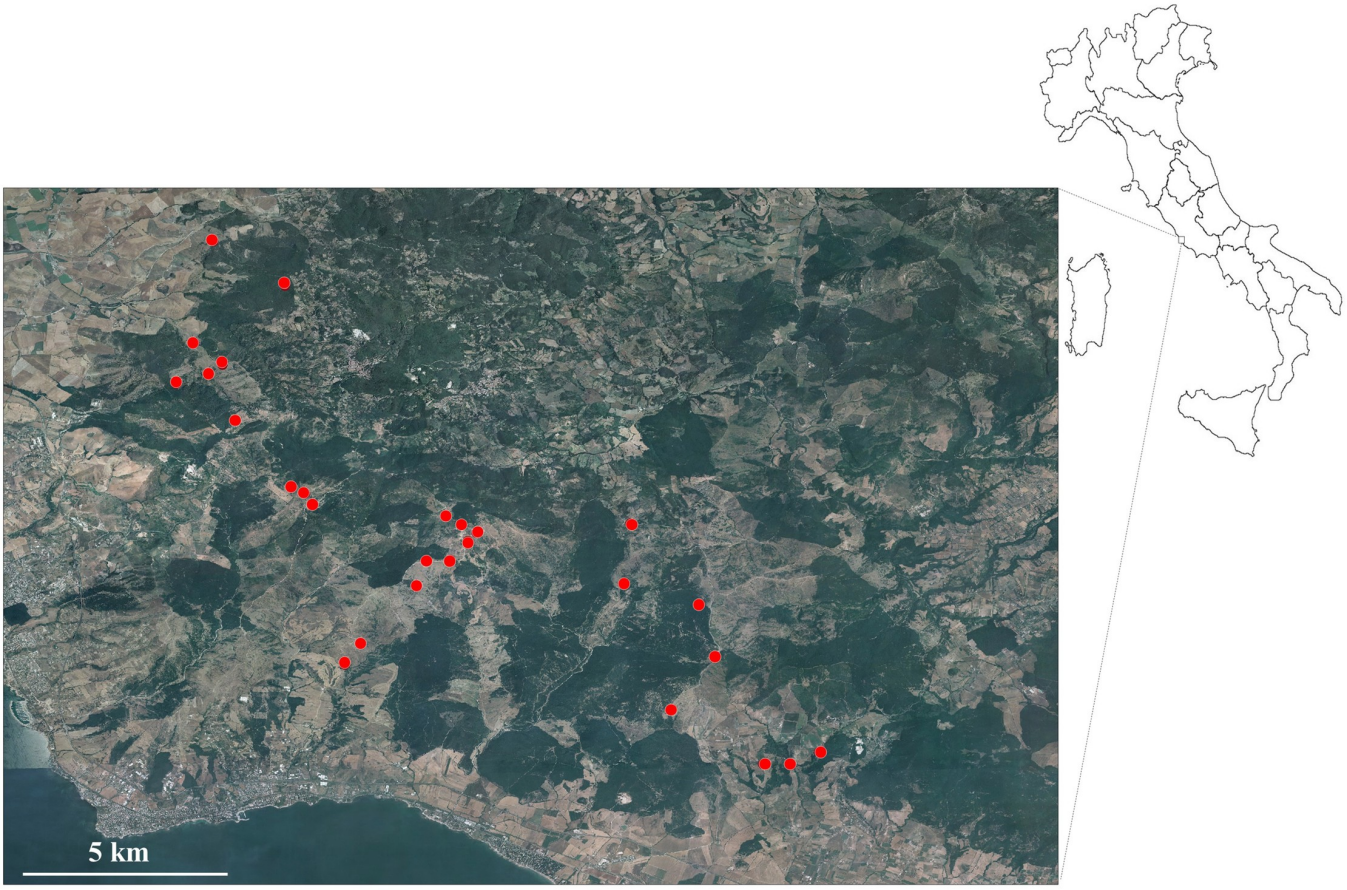
Map of the study area. The figure shows the area where the study was carried out and the sampled sites where Odonata were found. (Geographic photos with open access from site: http://cartografia.regione.lazio.it/cartanet/catalogo/catalog?folderinside=ortofoto#.W-Q_cZNKjcc; Legge Regionale n°12 del 10 agosto 2016).

**Fig 2:**
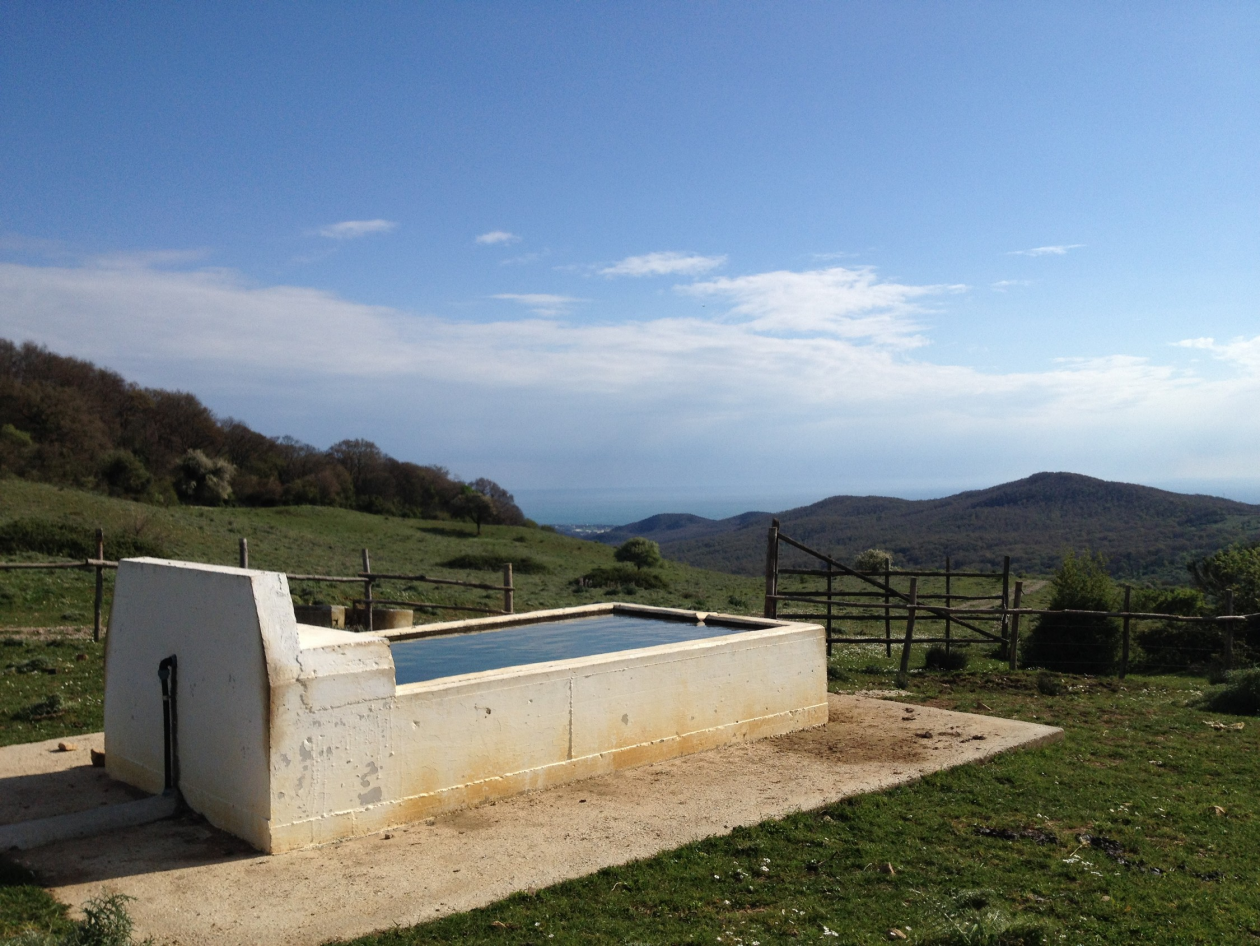
Drinking trough. Example of a trough in the study area.

We surveyed 37 drinking troughs dispersed over an area of about 8000 hectares (Fig 1). The drinking troughs in this area are brickwork tanks 4–10 m long, 1–1.5 m wide, 0.5–0.6 m deep, used as water reserve for free-range cattle, filled with running water deriving from springs and often kept full all over the year (Fig 2). Each drinking trough was characterized by taking note of the percentage of emerging, floating and submerged vegetation in relation to the volume (for the submerged vegetation: percentage of space of the water body occupied by vegetation) and to the surface (for floating and emerging) [4]. To collect Odonata species in the drinking throughs, we sampled the water body space by running nets (60x50x50 cm; 6 mm mesh) along the length of the entire trough. All the collected detritus and algae were inspected, and all macroinvertebrates were hand-collected and put in plastic boxes. The sampling was repeated three times per site (three immediately repeated nets run). All Odonata larvae were counted, measured and identified to estimate the relative abundance of the species. The specimens impossible to identify in the field were collected and fixed in alcohol 95% for microscope analysis and identification by using the most updated and comprehensive key identification guide for Italian dragonfly species [17]. The sampling period lasted from February to June 2017; collection of exuviae was also performed (in June) to avoid the bias due to some species whose occurrence could have been underestimated due to their occurring at the study sites as overwintering eggs.

### Statistical analyses

Data were organized in presence/absence matrices, and we performed null model analyses by measuring the Stone and Roberts' C-score as co-occurrence index [18]. It is a powerful index to detect nonrandom patterns in real matrices, even if they do not display a perfect checkerboard distribution for particular pairs of taxa [19]. We recall that the C-score measures the average number of "checkerboard units" (any submatrix of the form $\left(\begin{array}{cc} 1 & 0\\ 0 & 1 \end{array}\right)\;or\;\left(\begin{array}{cc} 0 & 1\\ 1 & 0 \end{array}\right)$) between all possible pairs of species occurring at least once in the matrix; in competitively structured assemblages, real communities should contain larger C score than randomly assembled communities [20]. We performed the analysis using the standard co-occurrence and the guild-structure modules of the software EcoSim (7.71 version) [21], maintaining the standard and suggested constraints of null matrices generation (fixed-fixed) where the observed row and column totals are maintained in the simulation and the number of occurrences of each species and the number of species in each site in the null communities are the same as in the original data set. 5000 initial swaps randomized the original pattern, yielding one random template matrix followed by 5000 consecutive swaps to create 5000 unique null matrices and the C-score was calculated for each simulated matrix. The observed C-scores were then tested against that of the simulated assemblages by evaluating the rank of observed values: an observed rank of less than 250 (i.e., 5% of 5000) or greater than 4750 (i.e., 95% of 5000) indicates statistically significant aggregation and segregation patterns among taxa, respectively, at p = 0.05.

After preliminary analyses, we decided to exclude Zygoptera species because each species occurred in just one site and always as singletons, from now we will refer only to dragonflies.

We tested by means of General linear model (GLM; link function: Log) the effect of some environmental variables of the drinking troughs and the degree of trough isolation on dragonflies community richness; we selected (i) aquatic vegetation (% volume), (ii) surface (drinking trough area, m^2^), and (iii) isolation (distance to the next closest drinking trough, m) as independent variables, and species richness (number of species of dragonflies) as dependent variable (Poisson distribution). The independent variables did not show collinearity (for all tests: r ≤ 0.216; p ≥ 0.234) and data did not shown overdispersion (c = -0.453, z = -0.408, p = 0.658, Dispersion test). The model included all the effects of independent variables and their interactions.

### Guild analyses

#### Guild membership

Ecological guilds represent groups of species within a community that share common resources or traits [22]. Species within a guild may be more likely to interact or compete for resources than are species in different guilds. We followed Corbet [5] for species belonging to distinct ecological guilds based on microhabitat occupancy by larvae that primarily differ in how they use legs to secure a resting position. In our assemblage, we recognized species belonging to the following guilds [5]: four to burrowers, five to sprawlers and one to claspers (*Anax imperator* Leach, 1815) (Table 1). We experimentally tested four species (two burrowers and two sprawlers) for their guild membership by assessing if the species’ preferred the type of micro-habitat matched with the ecological guild assigned to the species by Corbet [5]. Species belonging to the sprawlers guild are expected to crawl and climb on the aquatic vegetation, hiding between the trunks and leaves. Species belonging to burrowers are expected to stay on the bottom and wall substrate or to burrow under the sand. All experiments were performed at the Department of Science, Roma Tre University, Lab of Zoology. Larvae used were sampled from distinct drinking troughs and housed in 22 liter plastic tanks (39x28x28 cm) under a 12.12 h light:dark photoperiod, at an environmental temperature (18±2°C); animals were fed *ad libitum* with crustacean and Ephemeroptera larvae. The experiments were performed in identical plastic tanks with gravel sand as the bottom substrate, and aquarium plastic plants (17x20cm) were added to create vertical habitat structure. Five larvae of each species were introduced into the aquarium in the morning (11:00 a.m.). The number of larvae hidden or climbing on the artificial plant and burrowed or resting on the bottom was noted the next morning (24 h) [23].

**Table 1:** List of Dragonfly species collected during the surveys and description of guild where they belong. Species collected are grouped in ecological guilds. For each guild, we provided a brief description of the main features following Corbet [5].

Species	Guild	Guild description
*Anax imperator*	Claspers	Move readily and rapidly, feed in search mode, achieve crypsis by color and pattern, weakly thigmotactic
*Libellula depressa*	Burrowers	Dorsoventrally flattened body, they cover themselves with detritus from the ground, stay at the bottom of waterbodies
*Orthetrum brunneum*		
*Orthetrum cancellatum*		
*Orthetrum coerulescens*		
*Crocothemis erythraea*	Sprawlers	Move into well illuminated environment near the water surface, among moss or upright macrophytes, remain immobile when disturbed
*Sympetrum fonscolonbii*		
*Sympetrum sanguineum*		
*Sympetrum striolatum*		
*Sympetrum meridionale*		

Eight replicates were carried out for each species. We sampled from various drinking troughs a total of ca. 200 larvae belonging to the following four species: *Crocothemis erythraea (*Brullé, 1832), *Sympetrum* spp., *Libellula depressa* Linnaeus, 1758, *Orthetrum cancellatum* (Linnaeus, 1758). Larvae of *Sympetrum* genus were impossible to identify at species level since they had been found at too early a stage [17]. Data were analyzed by mean of Generalized Linear Models (GLM; link function: Logit; distribution error: Binomial) at species levels, where the species was selected as independent variable (categorical factor). The binomial output of every individual (in the plant or not) at the end of each trial was used as dependent variable.

#### Co-occurrence guild analysis

Presence-absence analysis

To perform co-occurrence guild analyses, we used the guild-structure module of EcoSim software that allows testing for patterns among the guilds as a group: i.e. whether the mean co-occurrence index *among* guilds is larger or smaller than expected by chance. The variance of the co-occurrence index among guilds is also analyzed: i.e. an unusually large variance would mean differences in levels of co-occurrence of the species *within* the guilds, while an unusually small variance would mean that guilds are strikingly similar to one another in the level of co-occurrence observed. A random result for the variance means that the level of co-occurrence among guilds is about what would be expected if the species were randomly split into different guilds [21]. We set the C-score as the index of co-occurrence. We excluded claspers (n = 1) from the analyses because only guild composed by species number > 2 can be analyzed. We tested burrowers and sprawlers consisting of four (*L*. *depressa*, *Orthetrum brunneum* (Fonscolombe, 1837), *O*. *cancellatum*, *O*. *coerulescens* Fabicius, 1798) and five species (*C*. *erythraea*, *Sympetrum fonscolonbii S*élys-Longchamps, 1776, *S*. *sanguineum* Müller, 1764, *S*. *striolatum* Charpentier, 1840, *S*. *meridionale* Sélys-Longchamps, 1841), respectively.

Abundance analysis

Methodological developments have revealed that in some cases (i.e. fine spatial scale), community-level analyses of presence–absence data may be unable to discriminate between multiple co-occurrence patterns (i.e. segregated, aggregated or nested distributions) because they average the species-pair values across the whole community [16]. Moreover, Ulrich and Gotelli [24] demonstrated that abundance is a powerful metric for quantifying patterns of species segregation and aggregation. Therefore, since information on relative abundances are essential for defining a community or a group of interacting species, we integrated the analyses on presence/absence data at the fine scale (i.e. ecological guild) with analyses on abundance data. Indeed, the analysis of co-occurrence patterns conducted at the species-pair level rather than at the level of the entire community can differentiate between segregation patterns produced by competition between a pair of species resulting in competitive exclusion, and aggregation produced by positive interactions that allow species to consistently co-occur [16,25,26,27].

By restricting the level of analysis to couples of potentially interacting species we can understand the role of single species pairs interaction in the assemblages that showed non-random structure. We performed pairs analysis at the guild level (fine scale). The pattern of abundances across replicated assemblages may potentially provide a more complex and subtle signal of community assembly pattern than binary (presence/absence) matrix [24]. We used the software Pairs [28] which allows measuring the C-score between single species pairs using both presence/absence and abundance data. We ran the pairs analysis using C-score as index and the <rc> as randomization algorithm, resampling rows according to the observed species abundance distribution calculated from row totals of abundance (Ulrich, pers. com., 2017). All the couple of species showing an observed C-score in the highest 5% of simulated values (i.e. higher than expected by chance) were considered as significantly checkerboarded and thus interacting in some ways. For more details on how Pairs software works we remand to the software manual [28]. We then determined the percentage of significant species pairs in each guild by dividing the number of significant species pairs by the number of possible species pair combinations. We compare the relative intensity of potential competition based on differences in the percentages of significantly checkerboarded species pairs.

#### Intra-guild predation

Based on co-occurrence guild analysis results, we carried out laboratory experiments to test intraguild heterospecific interaction (i.e. predation) as a main driver of the assemblage structure within sprawlers. We tested the effect of (i) habitat structure and (ii) availability of supplementary food on the predation rate by *C*. *erythraea* on *Sympetrum* individuals. The instar of the larvae used for this experiment were the following: *C*. *erythraea* F0/F1 against *Sympetrum* spp. F3/F4, in order to mirror the size difference, we found at the beginning of spring at the sites where the species occurred syntopically (in the same site). For each trial, we tested 5 vs. 5 heterospecific individuals for 48 h, and five replicates were performed for each setting (microhabitat and food). At the end of each trial, all the surviving larvae were counted. We standardized the hunger levels of larvae by starving them for two days before experimentation. Larvae were never re-used in different trials. For the supplementary food supply treatment, we provided crustaceans (cladocerans and copepods) ad libitum. The experimental design was set as 2x2 environmental states and was analyzed by means of GLM (link function: logit; distribution error: Binomial). Habitat structure (binary variable, fixed factor) and supplementary food supply (binary variable, fixed factor) were selected as independent variables. The number of alive *Sympetrum* individuals observed at the end of each trial was used as the dependent variable.

## Results

### Composition and co-occurrence patterns in Odonata assemblages

Overall, we collected 3292 larvae from 28 out of 37 sampled drinking troughs (ninedrinking troughs were found empty or damaged). We found 13 species (10 Anisoptera, 3 Zygoptera) belonging to four families (Table 2), with Libellulidae as the most represented family. *L*. *depressa* was the most common species (21 presences), followed by *S*. *sanguineum* (17 presences).

**Table 2:** Species checklist, number of sites where they have been found, and total abundance (number of individuals for each species).

Family	Species	N° Sites	Total abundance
Lestidae	*Chalcolestes viridis/parvidens*	1	2
Coenagrionidae	*Ischnura elegans*	1	2
	*Erythromma lindenii*	1	1
Aeshnidae	*Anax imperator*	3	29
Libellulidae	*Libellula depressa*	21	1848
	*Orthetrum brunneum*	11	221
	*Orthetrum cancellatum*	1	3
	*Orthetrum coerulescens*	1	2
	*Crocothemis erythraea*	12	316
	*Sympetrum fonscolombii*	5	60
	*Sympetrum meridionale*	8	94
	*Sympetrum sanguineum*	17	390
	*Sympetrum striolatum*	7	213

Among the environmental variables, only the percentage of aquatic vegetation showed a positive effect on the species richness of troughs (Wald = 8.831, p = 0.003), while the other variables and their interaction did not show any effect (p ≥ 0.08 in all cases).

The co-occurrence analysis performed on the whole dataset showed a non-random pattern with species segregating among the study troughs (C-score_obs_ = 14.154; C-score_exp_ = 13.269, Var_exp_ = 0.081, p = 0.003). The observed pattern remained non-random also after the exclusion of singletons belonging to Zygoptera (Anisoptera: C-score_obs_ = 21.577; C-score_exp_ = 20.433, Var_exp_ = 0.165, p = 0.009).

### Guild membership

**Fig 3:**
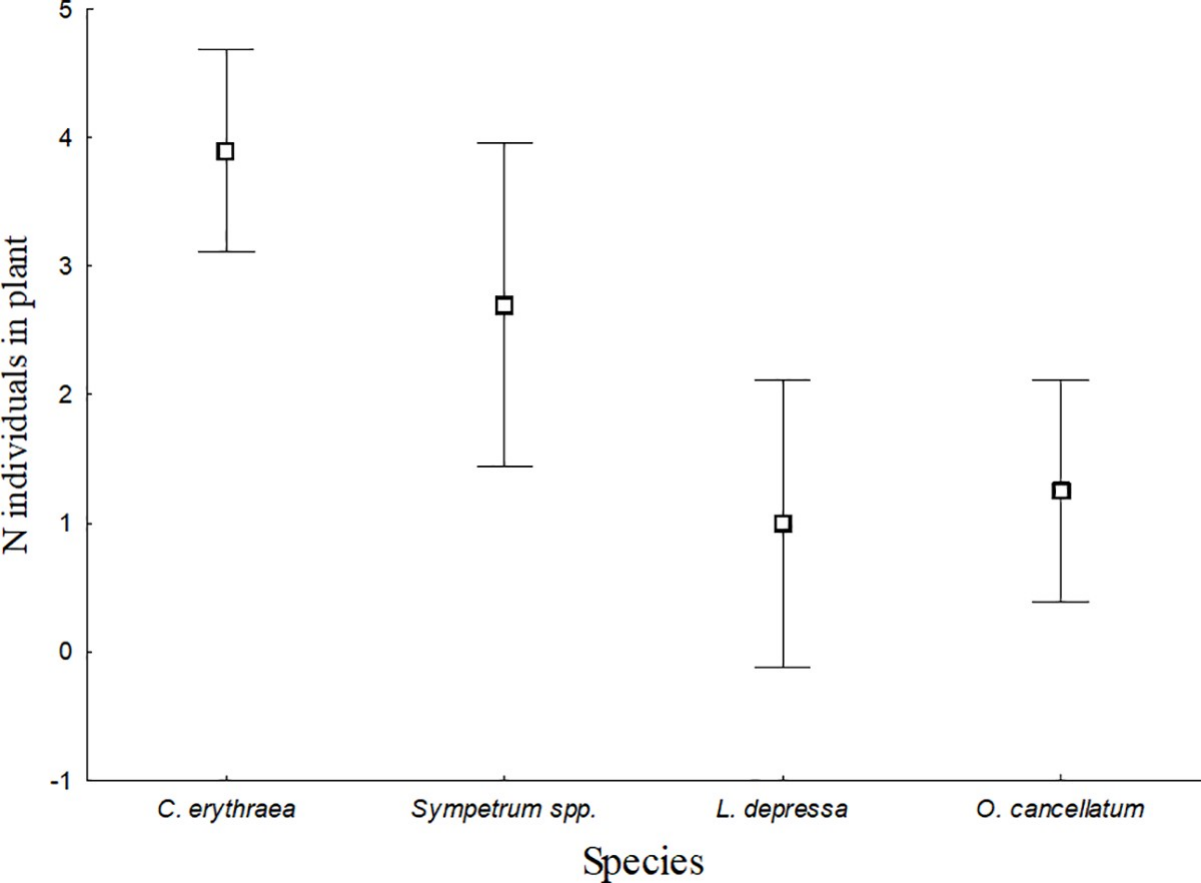
Use of plant by the species analyzed in the guild membership experiment. For each species, the number of individuals found climbing on plants is reported. Bars indicate 95% confidence intervals.

The number of larvae found climbing the plants was significantly higher for *C*. *erythraea* and *Sympetrum* (sprawlers guild) respect to *L*. *depressa* and *O*. *cancellatum* (burrowers). At the guild level, species behaved differently in habitat use (GLM: Wald = 32.129; p < 0.001), with sprawlers being found more frequently on plants than burrowers (for all post-hoc inter-guild comparisons p ≤ 0.001), whereas species belonging to a same guild did not differ (for all post-hoc intraguild comparisons p ≥ 0.060; Fig 3).

### Guild structure and analysis of species-pairs

There was no significant difference between observed and simulated C-score values (C-score_obs_ = 16.383, C-score_exp_ = 24.597, p = 0.086); hence, guilds seemed to not interact with each other in a competitive fashion. However, the guilds differed in the degree of intra-guild co-occurrence (observed variance significantly larger than expected, Var_obs_ = 525.961, Var_exp_ = 157.471, p = 0.043). Within each guild, burrowers showed random assembly (C-score_obs_ = 0.167, C-score_exp_ = 0.167, Var_exp_ = 0, p = 1), whereas sprawlers showed segregation (C-score_obs_ = 32.6, C-score_exp_ = 30.265, Var_exp_ = 0.554, p = 0.009). Within sprawlers, *C*. *erythraea* showed high numbers of checkerboard units (CU) calculated between each unique pair of species in the matrix, whereas among *Sympetrum* spp. on average CU values were lower (Table 3) Moreover, when the selected guilds were analyses by species-pairs with abundance data, nearly all the sprawlers’ species-pairs (80%) showed segregation (observed C-scores higher than simulated), with *C*. *erythraea* showing the higher values, together with *S*. *fonscolombii*, against the other sprawlers. (Table 4). As for burrowers, abundance data revealed that just 16% of species pair segregated (Table 4).

**Table 3:** Number of checkerboard units for all the sprawlers’ species pairs. “Checkerboard units" is any submatrix of the form $\left(\begin{array}{cc} 1 & 0\\ 0 & 1 \end{array}\right)\;or\;\left(\begin{array}{cc} 0 & 1\\ 1 & 0 \end{array}\right)$ between all possible pairs of species occurring at least once in the matrix. Average CU ± Standard Deviation for every species: *C*.*erythraea* 59.250 ± 15.041, *S*. *fonscolonbii* 30.000 ±13.089, *S*. *sanguineum* 18.500 ± 21.763, *S*. *striolatum* 28.500 ± 28.618, *S*. *meridionale* 26.750 ± 35.845.

Species	*S*. *fonscolonbii*	*S*. *sanguineum*	*S*. *striolatum*	*S*. *meridionale*
***C*. *erythraea***	44	50	66	77
***S*. *fonscolonbii***		13	35	28
***S*. *sanguineum***			11	0
***S*. *striolatum***				2

**Table 4:** C-score pairs analysis among the spralwers and burrowers guild species performed using abundance data. Sp: Species. S1 and S2: sites occupied respectively from species 1 and 2. Shared: number of shared sites. Obs. Score: Calculated C-score for the species pair. Exp. Score: Simulated C-score. In bold the significant species pairs.

Guild	Sp1	Sp2	S1	S2	Shared	Obs. Score	Exp. Score	p
Sprawlers	***S*. *sanguineum***	***C*. *erythraea***	**17**	**12**	**7**	**0.245**	**0.003**	< 0.001
	*S*. *sanguineum*	*S*. *meridionale*	17	8	8	0	0.007	1
	***S*. *sanguineum***	***S*. *striolatum***	**17**	**7**	**6**	**0.092**	**0.005**	< 0.001
	***S*. *sanguineum***	***S*. *fonscolonbii***	**17**	**5**	**4**	**0.153**	**0.01**	< 0.001
	***C*. *erythraea***	***S*. *meridionale***	**12**	**8**	**1**	**0.802**	**0.009**	< 0.001
	***C*. *erythraea***	***S*. *striolatum***	**12**	**7**	**1**	**0.786**	**0.004**	< 0.001
	***C*. *erythraea***	***S*. *fonscolonbii***	**12**	**5**	**1**	**0.733**	**0.014**	< 0.001
	*S*. *meridionale*	*S*. *striolatum*	8	7	6	0.036	0.01	1
	***S*. *meridionale***	***S*. *fonscolonbii***	**8**	**5**	**1**	**0.7**	**0.033**	< 0.001
	***S*. *striolatum***	***S*. *fonscolonbii***	**7**	**5**	**0**	**1**	**0.016**	< 0.001
Burrowers	***L*. *depressa***	***O*. *brunneum***	**21**	**12**	**11**	**0.04**	**0.000**	< 0.001
	*L*. *depressa*	*O*. *coerulescens*	21	1	1	0	0.017	1
	*L*. *depressa*	*O*. *cancellatum*	21	1	1	0	0.016	1
	*O*. *brunneum*	*O*. *coerulescens*	12	1	1	0	0.098	1
	*O*. *brunneum*	*O*. *cancellatum*	12	1	1	0	0.088	1
	*O*. *coerulescens*	*O*. *cancellatum*	1	1	0	1	0.705	1

### Intra-guild predation

**Fig 4:**
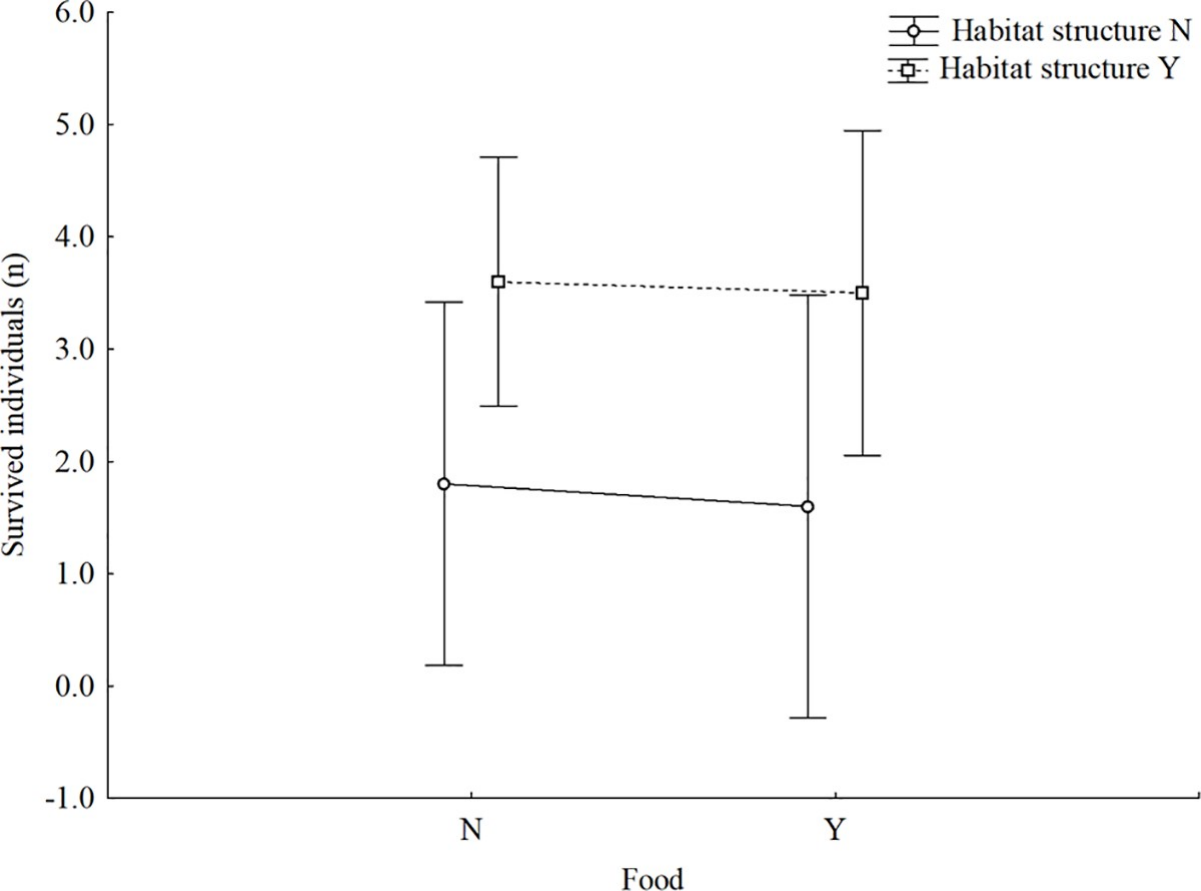
Interaction of habitat structure and food supply on the number of survived *Sympetrum* individuals. The model tested the influence the interaction of food supply and habitat structure on the interspecific predation between the sprawlers *C*. *erythrea* and *Sympetrum* spp.; Y = presence of artificial plant or alternative food, N = absence of artificial plant or alternative food. The bars represent the 95% confidence intervals.

Predation events were observed as a result of the interaction among different sized larvae (large *C*. *erythraea* vs. small *Sympetrum* spp.). Predation of at least one *Sympetrum* individual occurred in 18 of 20 trials. No intraspecific predation (i.e. cannibalism) was recorded. The presence of habitat structure (plant) influenced positively the number of surviving *Sympetrum* individuals (Table 5), whereas the presence of alternative food supply did not show any effect, as well as the interaction habitat*food (Fig 4).

**Table 5:** The effect of predation by *C*. *erythraea* on *Sympetrum* spp. GLM (Distribution: Binomial; Link function: Logit) results for the effect of habitat structure, food supply, and the interaction of factors on number of *Sympetrum* individuals survived at the end of predation experiments. Significant effects are in bold.

Effect	Wald	df	p
Intercept	0.05	1	0.823
Food	0.411	1	0.521
**Habitat structure**	**11.127**	**1**	**0.001**
Food*Habitat structure	0.05	1	0.823

## Discussion

### Composition and co-occurrence patterns in Odonata assemblages

We found that dragonflies assemblages living in simplified artificial habitats are non-randomly structured. This pattern can be interpreted as the result of interspecific interactions (i.e. competition and predation) that shaped local communities in terms of species composition and abundance [20]. Moreover, the intensity and importance of competition should increase as species become more similar in their ecological requirements [2,29]. Indeed, sprawlers were less likely to co-occur, suggesting an important role for competition in structuring intra-guild dragonfly communities. However, other factors (i.e. environment) can contribute in generating such a checkerboard pattern structuring the studied dragonfly communities. In the study system, drinking troughs shared similar size and depth and showed very low habitat heterogeneity with the only positive effect of aquatic vegetation coverage on local species richness. Therefore, environmental factors (i.e. trough size, location, surrounding habitats, etc.) should have a marginal or zero effect on assemblage composition. The hypothesis that the community structure is truly produced by species interactions and not by habitat segregation is thus reinforced by the fact that the strongest segregation patterns was observed among species with same habitat requirements [20]. Our explanation regarding the uncovered co-occurrence patterns is that species interact in some way at some life stages (by competing with and/or preying on other species) [30]. Focusing on larval life stages, the presence of structured assemblages can be expected due to the very simplified habitat characterizing the drinking troughs (e.g. limited volume, simple vegetation structure and diversity). This condition should facilitate larval population growth close to the threshold at which density-dependent interspecific negative interaction (competition/predation) are likely to occur. Our preliminary field observation on the adult stage revealed that the various species successfully deposit eggs despite other species patrolling the site. This would indicate that species interaction at this stage does not have a key role in shaping the larval community inside the drinking through. Larval ecological requirements and interaction in Odonata are known to be the main forces in governing assemblage composition and abundance because of the greater longevity, the higher density potentially reached, and the higher mortality characterizing this developmental stage compared to the adult age [5,31].

### Guild structure, species pairs and interactions

We found an idiosyncratic pattern of segregation between and within the considered guilds. The lack of observed segregation between guilds, although marginally supported (i.e. p = 0.086), should account for difference in auto-ecology and microhabitat use by the species; this divergence may allow interspecific interaction avoidance. In fact, it is unlikely that species that stay almost motionless on the bottom or along the stone walls of the trough (burrowers) could encounter species that climb on aquatic plants or under floating algae (sprawlers).This was confirmed by our laboratory experiments where the species belonging to sprawlers (*C*. *erythraea* and *Sympetrum* spp.) were observed climbing on the plant much more than burrowers did [23]. Therefore, we considered the likelihood to be very low that negative interactions like competition or predation could represent real constraints in assemblage patterning. Coexistence of more species from different guilds should be favored in troughs with different micro-habitat, and this fits with our result of a positive effect of the aquatic vegetation on species richness. Abundant aquatic vegetation that represent spatial niche for sprawlers and claspers requires a good bottom substrate, where burrowers can hide [5], and it would therefore lead to a richer local community.

When the guilds were analyzed separately, the situation changed. The burrowers (four species) showed no structure. However, the presence/absence matrix with two out of four species occurring in just one location could have biased the analysis. Indeed, from the abundance data emerged a significant segregation pattern for at least the two widespread species, *Libellula depressa* and *Orthetrum brunneum*. These two species have a similar flight period, do not perform diapause as egg [32] and are likely to hatch and grow at the same time, so decreasing the probability of substantial difference in body length during larval phase. Although we cannot summon the ghost of competition past to explain the lack of segregation among burrowers guild as a whole (i.e. species could stably co-existing in the same habitats), at the same time we cannot exclude that the pattern emerged with abundance data may indicate strong competitive interaction in the present [33,34] between *L*. *depressa and O*. *brunneum*. Indeed, by sharing most of their ecological niche, these species are prone to compete and then segregate when their density and the resource availability in troughs reach critical thresholds. We think that further investigation is needed to understand if burrowers are intrinsically characterized by an overall intra-guild stable competitive co-existence [34], or if a more pervasive segregation pattern among burrowers should have required more time to emerge than drinking trough history and management allowed.

The species belonging to sprawlers are clearly structured by biotic interactions in our drinking troughs, through larval assemblages. We note that intra-guild predation and competition can produce similar patterns among interacting species [35]. Our analyses supported that sprawlers segregated and their interactions should be considered among the main drivers that shaped the whole assemblage structure observed in our dragonfly communities. Our analyses on species-pairs overcame the inability to discriminate between multiple co-occurrence patterns when the entire assemblage is considered as a whole (i.e. by averaging the species-pair values across communities) and allowed to uncover segregation patterns at the very fine scale that remained otherwise neglected [16]. Specifically, within sprawlers, one species showed a higher rate of segregation (based on both checkerboard units and C-scores weighted with abundance data) when analyzed paired with the other sprawlers, and we recall that species pairs that did not show signals of significant environmental variation or dispersal limitation may be evidence of a significant species interaction [13]. The species, namely *Crocothemis erythraea*, can reproduce twice per year [32] and its first oviposition takes place in first summer. The remaining sprawlers (*Sympetrum* spp. group, containing *S*. *striolatum*, *S*. *sanguineum* and *S*. *meridionale*) are used to oviposit overwintering clutches, their diapause eggs hatching after a period longer than 80 days is usually correlated to the duration of winter [5]. This means that in spring, when *Sympetrum* larvae hatch, those of *C*. *erythraea* are already well developed and could prey on the newly hatched individuals. This scenario may have occurred at the sites shared by *C*. *erythraea* and *Sympetrum*. spp., with larvae of the former species ranging in length 1.2–1.9 cm (F0/F1 instar) and those of the latter ranging between 0.5–0.8 cm (F3/F4 instar).

By recreating the hetero-specific divergence in body size due to heterochronic development, our experiments demonstrated that intraguild predation, already studied in other dragonflies but not previously known for the study species [5,36,37] can be a main driver determining assemblage composition. Indeed, predation of small *Sympetrum* individuals by larger *C*. *erythraea* individuals occurred in all experimental settings, even in presence of alternative food supply. This would indicate that despite the availability of other prey in the drinking troughs, the asymmetrical intraguild predation remains an important trophic interaction. Indeed, dragonfly larvae may represent an elective prey for other species, especially for other Odonata belonging to the same guild, due to the very high population density reached in a single trough (over 400 individuals) and to the ease of the encounter rate due to the shared microhabitat [38]. Moreover, our laboratory results confirmed that the presence of aquatic vegetation could be a factor enhancing the likelihood of species syntopy (that we found in some troughs) by providing habitat structure with places to hide for the young (and smaller) larvae; indeed, this is in agreement with the observed positive effect of aquatic vegetation on trough species richness.

Overall, our results demonstrated that the time of hatching may be of particularly importance in structuring dragonfly assemblages because asynchronous development may facilitate the onset of negative interactions [39,40]. Anholt [39] found that survival of early-instar *Enallagma boreale* (Selys, 1875) may be 50% in the absence of larger conspecifics, but only 3% in their presence. Hence, large or fast-growing larvae are superior predators to small or slow-growing larvae with the former influencing the abundance of the latter [40].

As for the temporal horizon within which such patterns may emerge, we must consider that: (i) the larval cycle of the studied species spans only few months, and (ii) drinking troughs often undergo water regime instability (i.e. completely dry up in summer season) and cleaning. Contrarily to most ecological processes that require long-term coexistence to produce structure in assemblages, the observed ecological pattern must be generated by mechanisms that shape the community in the very short-term. We thus hypothesize that the structure based on interspecific interactions found in our dragonfly assemblages emerged in the very short term, at the order of few years, or just one season.

We would like to emphasize that our field study, although cannot demonstrate that competition has occurred, identified patterns that are compatible with the effects of competitive interactions [16]. The observed Odonata assemblage structure is consistent with the interpretation that processes (species exclusion) based on strong biotic interactions (i.e. competition or predation) has shaped the distribution of the Odonata species under consideration and represents the starting point for testing which mechanism could have produced it. Our manipulative experiments represent the conclusive evidences [34] demonstrating that Odonata intra-guild predation is reasonably at least one of the main determinants of a segregation patterns based on species exclusion.

## Conclusion

The ‘checkerboard arrangements’ resulting in low co-occurrence patterns may correspond to several processes such as: (1) ‘historical checkerboard’ that corresponds to forbidden species pairs caused by different evolutionary or biogeographical histories; (2) ‘habitat checkerboard’ that results from differential affinities for non-overlapping habitats between species; (3) ‘stochastic checkerboard’ arising e.g. from random local extinction and re-colonization events; and (4) ‘competitive or ecological checkerboard’ observed when species pairs are impeded by interspecific interaction exclusion [41]. Clearly the first two process are not to be considered in our study: the geographical scale is too small to claim biogeographical issues behind our patterns; we consider unlikely an effect of ‘habitat checkerboard’ because the species considered here inhabit lentic waters and are widespread over the entire study area with a large overlap among drinking troughs. A ‘stochastic checkerboard’ effect cannot be excluded since the drinking trough management (deep cleaning) can cause the complete extirpation of a local community with obvious effects on community assembly. However, this effect could have had a minor influence on the overall pattern because drastic cleaning procedures occur at low frequency and do not involve all the drinking throughs. Instead, we demonstrated that the co-occurrence patterns in the study system are mainly caused by interspecific interactions. Predation between larvae at different development stages may result in an effective segregation pattern, representing one of the several processes that concur in producing such patterns in dragonfly assemblages.

## Supporting information

S1 File Species matrices and sites coordinates. The S1 File contains three sheets: 1) the presence/absence matrix for all Odonata (Zygoptera and Anisoptera) species in the drinking troughs; 2) two matrices for the two guilds (sprawlers and burrowers) with species abundance data in every site; 3) GPS coordinates of drinking troughs sampled.
(XLSX)
